# Wind Tunnel Measurement of Turbulent and Advective Scalar Fluxes: A Case Study on Intersection Ventilation

**DOI:** 10.1100/2012/381357

**Published:** 2012-01-16

**Authors:** Libor Kukačka, Štĕpán Nosek, Radka Kellnerová, Klára Jurčáková, Zbyněk Jaňour

**Affiliations:** ^1^Department of Meteorology and Environment Protection, Faculty of Mathematics and Physics, Charles University in Prague, V Holešovičkách 2, 180 00 Prague, Czech Republic; ^2^Institute of Thermomechanics AS CR, v.v.i., Dolejškova 1402/5, 182 00 Prague, Czech Republic

## Abstract

The objective of this study is to determine processes of pollution ventilation in the X-shaped street intersection in an idealized symmetric urban area for the changing approach flow direction. A unique experimental setup for simultaneous wind tunnel measurement of the flow velocity and the tracer gas concentration in a high temporal resolution is assembled. Advective horizontal and vertical scalar fluxes are computed from averaged measured velocity and concentration data within the street intersection. Vertical advective and turbulent scalar fluxes are computed from synchronized velocity and concentration signals measured in the plane above the intersection. All the results are obtained for five approach flow directions. The influence of the approach flow on the advective and turbulent fluxes is determined. The contribution of the advective and turbulent flux to the ventilation is discussed. Wind direction with the best dispersive conditions in the area is found. The quadrant analysis is applied to the synchronized signals of velocity and concentration fluctuation to determine events with the dominant contribution to the momentum flux and turbulent scalar flux.

## 1. Introduction

Dispersion of air pollution within urban areas is an important aspect of the environment quality for a significant part of the population. Vehicle emissions represent the main source of pollutants in large cities, Fenger [1], Colvile et al. [2]. The dispersion in street canyons determines a spatial distribution of pollutants and their dangerous impact. Short-time average concentrations measured especially in lower parts of the street canyons often reach threshold values. Improvement of air quality in urban areas is necessary to avoid risk for human health, Hoek et al. [3], Nyberg et al. [4].

We can define ventilation of an urban area as a process of changing polluted and fresh air within street canyons, which improves the air quality. Ventilation is directly caused by horizontal and vertical transport of pollution out from the area.

Wind tunnel investigations provide an environment where flow and dispersion can be explored in relatively stationary conditions and allow facile changes of model geometry. Several wind tunnel studies focused on concentrations within canyon for a tracer emitted at street level and flow perpendicular to the street, Kastner-Klein and Plate [5], Pavageau and Schatzmann [6]. The transport of pollution to the air above the roof level was estimated from measurements of concentrations in these works.

Wind tunnel and field studies for relatively symmetrical and regular street canyons arrangements express influence of geometry of streets and intersections in pollutant dispersion and hence ventilation in urban areas, see Brown et al. [7].

Mixing and transport processes in a simple street and its ventilation were elaborated by Belcher [8]. In this work ventilation fluxes were determined for estimation of the mean scalar transport within the urban street network. Barlow and Belcher [9] focused on studying the ventilation characteristics of a street canyon for the simple case of wind perpendicular to the street. Wind tunnel experiments published by Robins [10] show that the mass exchange between street canyons may be significantly changed due to a small variations of the building geometry. These results were obtained from computing scalar fluxes determining pollution transport. Results from numerical simulation published by Scaperdas and Colvile [11] show a very complex behaviour of the flow in an urban area. This work shows configuration of the street canyon and the wind direction when air exchange between alongwind and crosswind streets is reversed. Numerical and wind tunnel simulations of the flow and dispersion near regular and irregular street intersections were studied by Wang and McNamara [12].

Presented papers demonstrate high sensitivity of flow and dispersion processes to the intersection geometry and wind direction that are naturally connected with ventilation of an urban area.

Several publications have been focused on the air quality near street intersection in detail, for example, Dabberdt et al. [13]. Significantly higher pollution concentrations have been observed near intersections than along the streets with a continuous traffic, see Claggett et al. [14]. The reason of this observed phenomenon is that vehicles spend longer period of time near junctions, in driving modes that generate more pollutants (decelerating or accelerating), than in relatively steady movement in streets. The review of the traffic pollution modeling was published by Sharma and Khare [15].

The objective of this study is to determine processes of the traffic pollution transport within the X-shaped intersection in an idealized symmetric urban area for several approach flow directions. Pollutant is emitted into the urban area from the point source simulating “pollution hotspot”—the place with higher emission of traffic pollution situated near a junction, Soulhac et al. [16], Tomlin et al. [17].

## 2. Experimental Setup

### 2.1. Wind Tunnel

**Figure 1:**
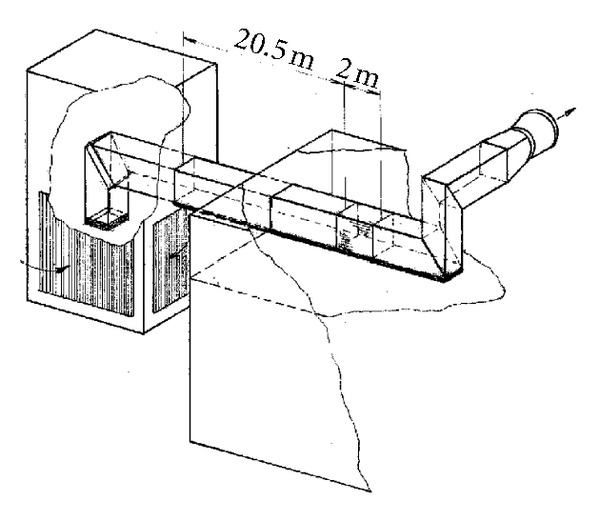
The scheme of the open low-speed wind tunnel.

 The experiment was conducted in the open low-speed wind tunnel of Institute of Thermomechanics Academy of Sciences of the Czech Republic in Nový Knín. The crossdimension of the tunnel test section was 1.5 × 1.5 m, the length of the test section was 2 m. The scheme of the tunnel is depicted in Figure 1.

**Figure 2:**
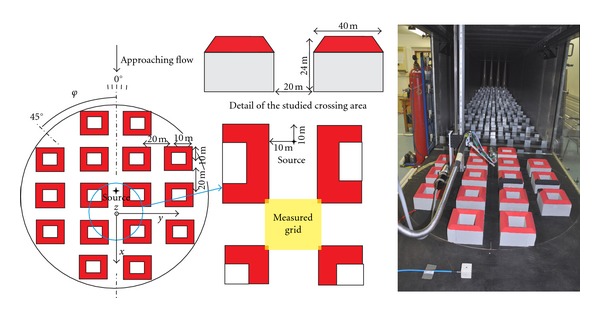
Scheme of the idealized symmetric urban area model (left), the studied X-shaped intersection (middle), and the photograph of the model placed in the wind tunnel (right).

Fully turbulent boundary layer was developed by the 20.5 m long development section of the tunnel. This section was equipped by turbulent generators at the beginning and covered by 50 mm and 100 mm high roughness elements on the floor, see the photo in Figure 2.

### 2.2. Urban Area Model

 The model of idealized symmetric urban area with apartment houses was designed according to the common central European inner-city area. Regular blocks of apartment houses with pitched roofs formed a perpendicular arrangement of the street canyons and X-shaped intersections, see Figure 2.

The model was scaled down to 1 : 200. The model buildings were formed by the body of height 100 mm and width 50 mm with pitched roof of height 20 mm. We setup the characteristic building height *H* = 120 mm (24 m in full scale) as the height of building body with the roof.

The width of street canyons was *L* = 100 mm. The aspect ratio of the street canyons given by the building height *H* and the street width *S* was *H*/*L* = 1.2.

A point pollution source simulating a “pollution hotspot” (the place with higher emission of traffic pollution situated near a junction) was placed at the bottom of the street canyon in front of the studied intersection, see Figure 2.

### 2.3. Measurement Techniques

 The flow characteristics were measured using a two-dimensional optical fibre Laser Doppler Anemometry (LDA), based on DANTEC BSA F-60 burst processor. Tracing particles (glycerine droplets with approximately 1 *μ*m diameter) were produced by a commercial haze generator placed at the beginning of the tunnel generating section, in front of turbulent generators. We got the air flow in the test section equally filled by seeding particles after running the haze generator inside the tunnel for several minutes. Data rate reached about 100 Hz at the bottom levels of street canyons *z*≲0.5*H* and up to 1000 Hz at the roof top level *z* ≈ *H*. The time of recording was 180 s in all the cases.

Point concentration measurements of tracer gas were realised by Slow-Response Flame Ionisation Detector (SFID) and Fast-Response Flame Ionisation Detector (FFID). We used SFID (type ROSEMONT NGA 2000) for mean concentration measurement within the studied intersection. Simultaneous vertical velocity and concentration measurement at the roof top level above the intersection were realised using LDA and FFID (type Cambustion Ltd. HFR400 Atmospheric Fast FID). The FFID was set to acquire data at a data rate of 1 KHz. The sampling time was 180 s in all of the cases.

We used ethane as the tracer gas simulating passive pollutants. Ethane is passive and nonreactive gas with its own density *ρ*_Ethane_ = 1.24 kg · m^−3^ close to density of the air *ρ*_Air_ = 1.28 kg · m^−3^.

SFID and FFID were calibrated approximately every four hours of measurement. The differences in output voltage reached up to 5% through the measuring campaign. All the concentration values were computed from measured voltage signal using linear interpolated values from two calibrations realised before and after the recorded data set.

We applied a standard three-point calibration for the SFID measurement using clean air (air sucked into the wind tunnel from the atmosphere) and two span gases of known hydrocarbons concentrations.

For simultaneous velocity and concentration measurement, the four-point FFID calibration using clean air (air sucked into the wind tunnel from the atmosphere), air equally filled by seeding particles, and two span gases of known hydrocarbons concentrations was obtained.

As expected, the presence of the seeding particles in the air during simultaneous LDA and FFID measurement influenced FFID output signal. At first we got isolated spikes in the recorded concentration signal probably due to suction of combustible aerosol particles from air into the FFID probe. The problem was mentioned by Hall and Emmott [18], Contini et al. [19]. Unlike these published results, we got similar count of spikes in time series obtained from measurements in clean air and in air contained seeding particles in most cases. We neglected the influence of spikes on the results because the frequency of isolated spikes was about 0.006% of used sampling data rate.

The second influence of seeding particles on the measured concentration data was an almost constant shift of recorded concentration values caused obviously by sucking seeding particles by FFID probe. This shift reached about 0.5% of the FFID measuring range. The shift was corrected by the calibration sequence mentioned above.

**Figure 3:**
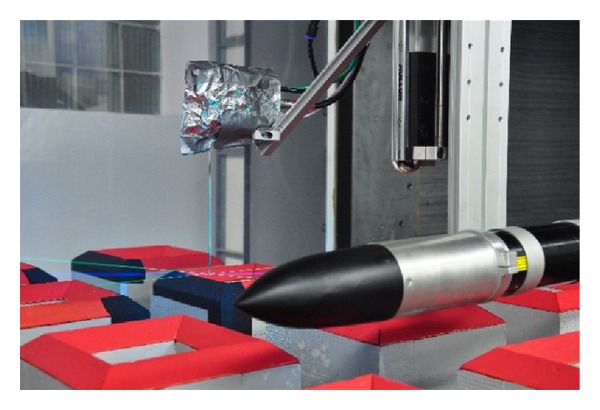
The configuration of the FFID (left) and LDA (right) probes mounted on the traverse system in the wind tunnel.

For simultaneous velocity and concentration measurement, LDA and FFID probes were mounted on the traverse system in a way that the measuring volume of the LDA was close to the intake of the FFID sampling tube. The sampling tube intake was placed 1.5 mm above, 1 mm behind, and 1 mm beside the centre of the LDA measuring volume. Several test measurements with different positions of both probes demonstrated a negligible influence of FFID sampling tube placed close to the LDA measuring volume on the flow. The configuration of probes is captured in Figure 3.

### 2.4. Boundary Layer Characteristics

**Figure 4:**
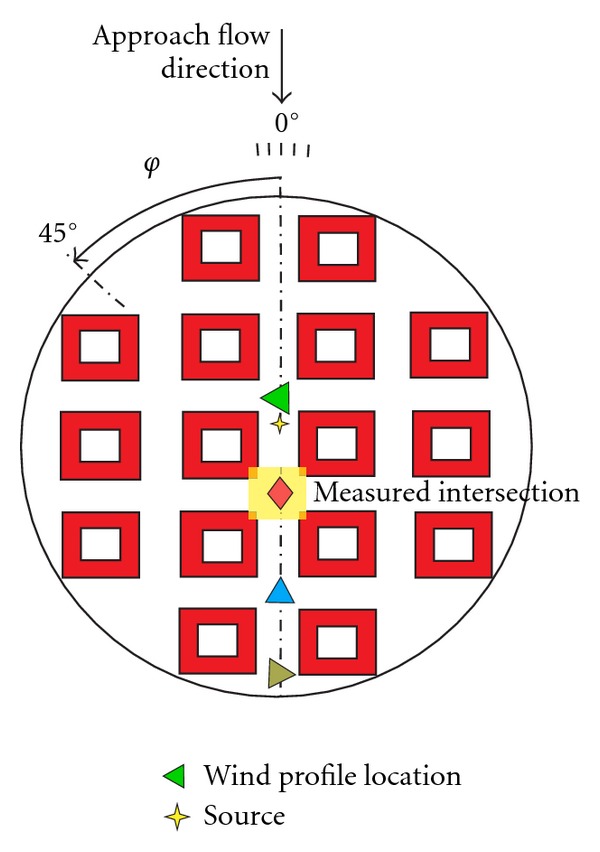
Wind profile measurement locations.

 Fully turbulent boundary layer was developed by spires and roughness elements placed it the tunnel. The characteristics of the boundary layer above the urban area model were measured with a two-dimensional LDA system in four vertical profiles placed above, upstream, and downstream from the studied intersection, see Figure 4.

**Figure 5:**
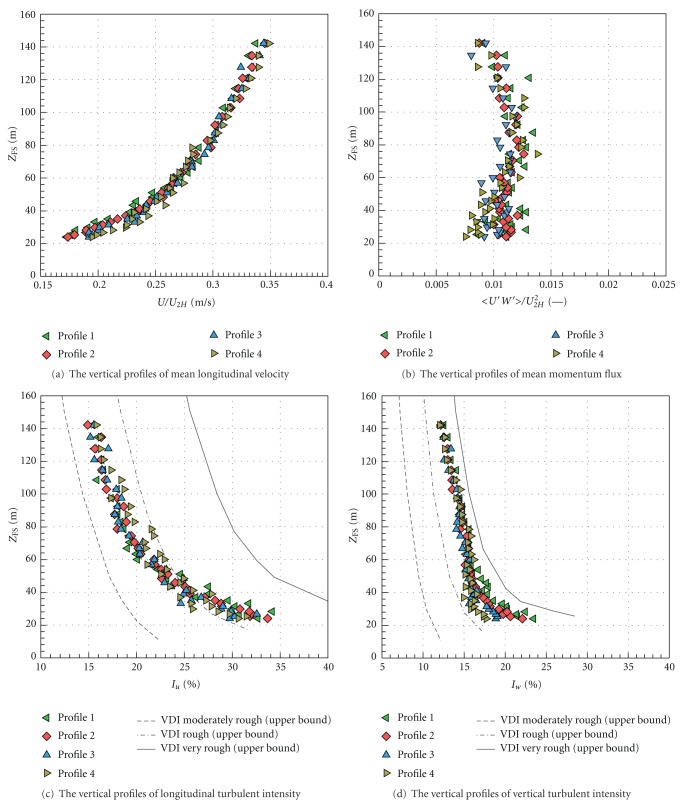
Boundary layer characteristics above the urban area model.

The vertical profile of mean longitudinal velocity is depicted in Figure 5(a), the momentum flux profile can be found in Figure 5(b). The vertical profiles of longitudinal and vertical turbulent intensity are plotted in Figures 5(c) and 5(d). The high above the surface is expressed in full scale.

Vertical profiles of measured turbulent approach flow characteristics were fitted by the logarithmic and the power law. Mean roughness length *z*_0_, displacement *d*_0_, and friction velocity *u*_∗_ (alias square-root of constant value of Reynolds stress within the inertial sublayer) were obtained from the logarithmic fit. Power exponent *α* was obtained from the power fit. The parameters are listed in Table 1.

Categories of boundary layer are defined according to classification in VDI [20]. Measured parameters corresponded to a neutrally stratified boundary layer flow above a densely built-up area without much obstacle height variation.

To verify requirements for the Townsend hypothesis, see Townsend [21], the critical Reynolds building number *Re*_*B*_ was found. This criterion was used by Meroney et al. [22] and Pavageau and Schatzmann [6] for the flow within street canyons to be independent of viscous effects. The Reynolds building number modified for our experiment was given by

(1) ReB=U2HHν,

where *ν* is kinematic viscosity. The experiment was carried out by *Re*_*B*_ ≈ 21000 that lies on the lower edge of determined interval for valid Townsend hypothesis. Free stream velocity was approximately 4 ms^−1^.

## 3. Results

 Horizontal velocity of the flow and concentration of the tracer gas was measured in vertical cuts (cross-sections) labelled A, B, C, and D. These cuts were placed in the exit planes of the street canyons connected to the studied intersection, see Figure 2. Cuts were placed 5 mm inward to the canyons because of the high gradients of measured quantities and the strongly unstable flow at the exact exit planes of the street canyons.

Furthermore, the vertical velocity and tracer gas concentration were simultaneously measured in a horizontal plane at the roof level *z* = *H* above the studied intersection. We used a reference velocity *U*_2*H*_ measured at the reference height *z* = 2*H*. Results were obtained from five different values of the approach flow angle *φ* = 0°, 5°, 15°, 30°, and 45°.

**Figure 6:**
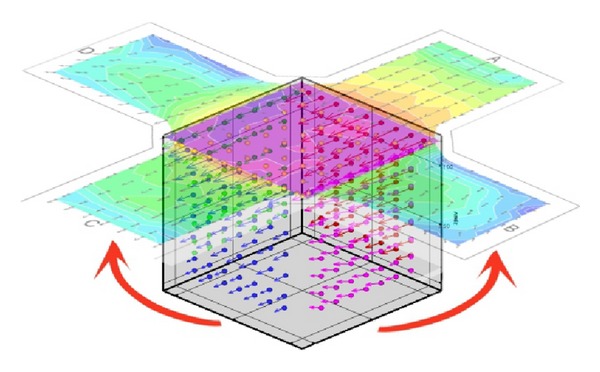
The transformation of the measured three-dimensional grid to an horizontal plane.

In order to get an understandable image of the results, we used a transformation of the measured three-dimensional grid to a horizontal plane, see Figure 6. Vertical cuts of the measured grid were tipped out to the horizontal plane given by the roof level of the intersection. An orientation of horizontal velocity vectors in the vertical cuts was maintained in the transformed horizontal plain image.

### 3.1. Mean Velocity Fields

 The flow inside the canopy was strongly three-dimensional and vortices of various scales are formed within and above the canyons and intersections. Measured components of velocity vector are expressed by the dimensionless form given by

(2) $$\frac{U}{U_{2H}},\;\;\frac{V}{U_{2H}},\;\;\frac{W}{U_{2H}},$$

where *U* and *V* are the horizontal velocity components measured in vertical cuts placed in the exit planes of the street canyons connected to the studied intersection, *W* is vertical velocity of the flow measured in the horizontal plane at the roof level *z* = *H* above the intersection. *U*_2*H*_ means a reference velocity measured at the reference height *z* = 2*H*.

A contour plots of velocity magnitude were added to the images of the velocity field. The orientation of horizontal velocity components is given by plotted vectors. The orientation of vertical velocity is given by a sign of the scalar values: the positive sign means an upward direction of vertical velocity and the negative sign means a downward direction.

**Figure 7:**
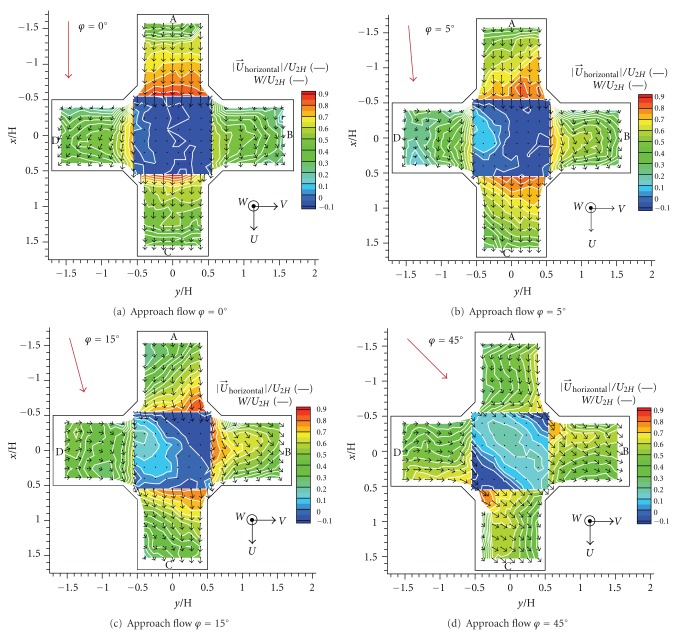
Dimensionless velocity fields for four angles of the approach flow direction.

A roughly symmetrical velocity field was formed by *φ* = 0° (Figure 7(a)). The main stream was situated to an alongwind street parallel with the approach flow (Cuts A and C). A vortex with vertical axis was formed within the crosswind streets (Cuts B and D). The horizontal velocity decreased in levels towards the bottom of the street canyons (further form the middle of the picture). The vertical velocity on the top of the intersection was negligible in this case.

We observed an obvious change in the velocity field by *φ* = 5° (Figure 7(b)). The main stream was still situated to an alongwind street, but the horizontal velocity increased in the left transverse street and decreased in the right transverse street. There was a small increase of upwards vertical velocity on the right side. A region with upward vertical velocity was formed near the right leeward corner.

As for the angle *φ* = 15° as well as *φ* = 30°, a significant stream was formed within crosswind streets (Figure 7(c)). The increase in the upwards vertical velocity continued on the right side; however, it was not so important as in comparison with the changes of the vertical velocity.

An almost symmetrical velocity field was formed by *φ* = 45° (Figure 7(d)). The main stream was divided into the alongwind and left crosswind streets (Cuts B and C). Asymmetry of flow was probably caused by minor geometrical deviations of the model in case of approach flow angle *φ* ≈ 0°.

### 3.2. Mean Concentration Fields

 The dimensionless concentration for a point source was obtained from the formula published in VDI [20]:

(3) $$C^{*}=\frac{CU_{2H}H^{2}}{Q},$$

where *C* means the measured concentration in and *Q* is a source emission volume flow.

**Figure 8:**
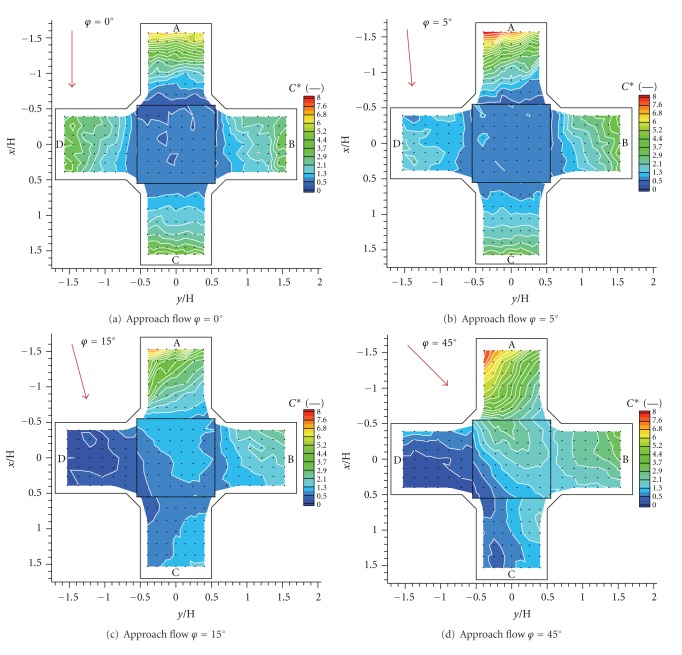
Dimensionless concentration fields for four angles of the approach flow direction.

Values of computed dimensionless concentration for five angles of the approach flow directions are plotted in Figure 8. A roughly symmetrical concentration field was formed by *φ* = 0° (Figure 8(a)), but notice slightly higher concentration in alongwind street (Cut D) in comparison with the left crosswind street (Cut B). There was almost zero concentration on the top of the intersection, which indicated weaker advective vertical transport of pollution than in the following cases.

We observed that a quite small change of the angle of the approach flow caused a radical change in concentration field by *φ* = 5° (Figure 8(b)). An obvious deformation of the concentration field is probably caused mainly by street canyon vortices with horizontal axis (Cut A). The decrease in concentration in the right crosswind street (Cut D) was measured.

Transport of the majority of the tracer gas from the alongwind street (Cut A) to the left crosswind street (Cut B) was obvious by *φ* = 15° (Figure 8(c)). Consequently there was almost zero concentration in Cut D. We measured the lowest concentrations in the intersection area in this case. We got a very similar concentration field for *φ* = 30° (not shown).

We observed an overall increase in concentration by *φ* = 45° (Figure 8(d)). There was an enhanced transport of the tracer gas to the right crosswind street up the approach wind (Cuts C and D) compared with case *φ* = 15°. It was probably caused by a small vortex with vertical axis at the leeward wall of this street. There was an area of significant concentration at the top of the intersection.

As we expected, the highest concentrations were measured at the ground levels in all cases and at the leeward wall of the street with the source (Cut A).

### 3.3. Advective Scalar Flux Fields

 The dimensionless advective scalar fluxes were computed from the average measured data to quantify advective spreading of pollutants within the studied intersection, see similar approach in Belcher [8], Robins [10]. We computed horizontal dimensionless advective fluxes using forms

(4) $$\frac{C^{*}U}{U_{2H}},\;\;\frac{C^{*}V}{U_{2H}},$$

where C* is the mean dimensionless concentration of the tracer gas, *U* and *V* are the mean horizontal velocity components of the flow. Vertical dimensionless advective flux given was given by

(5) $$\frac{C^{*}W}{U_{2H}},$$

where *W* is the mean vertical velocity of the flow. Results were obtained for all five values of the angle of the approach flow *φ* = 0°, 5°, 15°, 30°, and 45°.

The dimensionless advective scalar fluxes expressed a rate of emissions spreading through an unit area. Computed fluxes characterized the advective transport of pollution with the following convention of signs: the positive sign means the flux outwards and the negative sign means the flux inwards the studied intersection.

**Figure 9:**
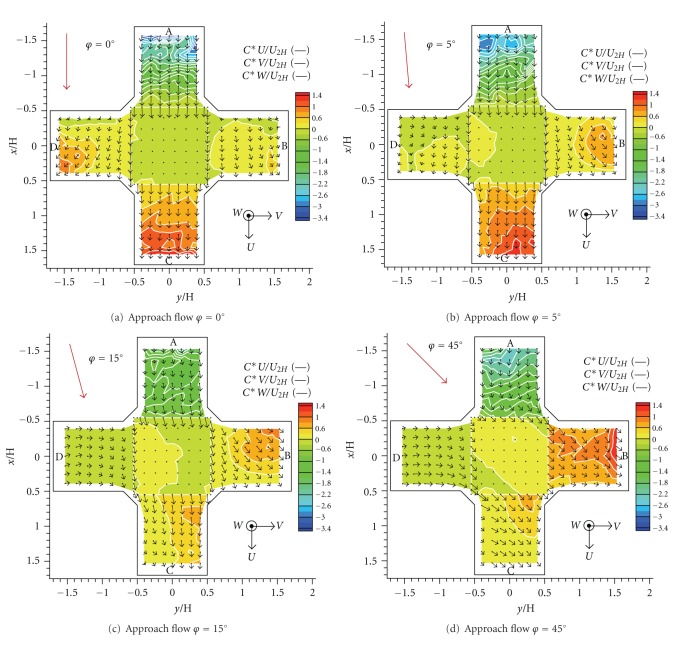
Horizontal and vertical dimensionless advective flux of passive contaminant with horizontal velocity vectors for four angles of the approach flow direction.

Values of computed fluxes for five angles of the approach flow directions are plotted in Figure 9. We can observe quite an asymmetrical flux field by *φ* = 0° (Figure 9(a)). There is a higher flux into the right crosswind street (Cut D) than into the left crosswind street (Cut B). As we mentioned this was probably caused by minor geometrical deviations of the model. However, it means very strong sensitivity of scalar fluxes to the geometry of the model and approach flow direction. Notice a negative, that is, downward, flux at the top.

A roughly reversely spread flux field was formed by *φ* = 5° (Figure 9(b)) compared to the case of *φ* = 0°. We could see a significant transport into the left crosswind street (Cut B).

A noticeable overall decrease in the flux in case of *φ* = 15° was observed (Figure 9(c)). The lowest fluxes were measured in this case within the studied area. Emissions were transported mainly to the left crosswind street (Cut B). There was an area of the positive flux on the right side at the top of the intersection. We got similar flux field for *φ* = 30° but with higher flux values.

A spreading of emissions mostly to the left side still predominated by *φ* = 45° (Figure 9(d)). There was an increase in the flux especially in the left crosswind street (Cut B). There was mostly a positive flux at the top of the intersection.

### 3.4. Turbulent Scalar Flux Fields

 The dimensionless vertical turbulent scalar fluxes were computed from synchronised vertical velocity and concentration signals using eddy-correlation method, Arya [23], Stull [24].

The used Matlab postprocessing script synchronised simultaneously acquired vertical velocity and concentration data using the maximum of correlation between both signals. The synchronised time series were shifted by an average of 15 ms. This shift expressed the delay between a suck of the sample into the intake of the FFID probe tube and the moment of the sample analysing in the probe. The value of the shift agrees with very similar experimental setup published by Contini et al. [19].

The dimensionless vertical turbulent scalar flux is given by

(6) $$\begin{array}{c} \frac{\langle c^{*\prime }w^{\prime }\rangle }{U_{2H}}, \end{array}$$

where 〈⋯〉 mean a time average, *c* ^∗′^ and *w*′ indicate fluctuations of dimensionless concentration and vertical velocity. Similar approach to turbulent transport computing was published in Jurčáková et al. [25].

Computed dimensionless vertical turbulent fluxes express a rate of emissions spreading through a unit area by turbulent transport with the same convention as mentioned above.

**Figure 10:**
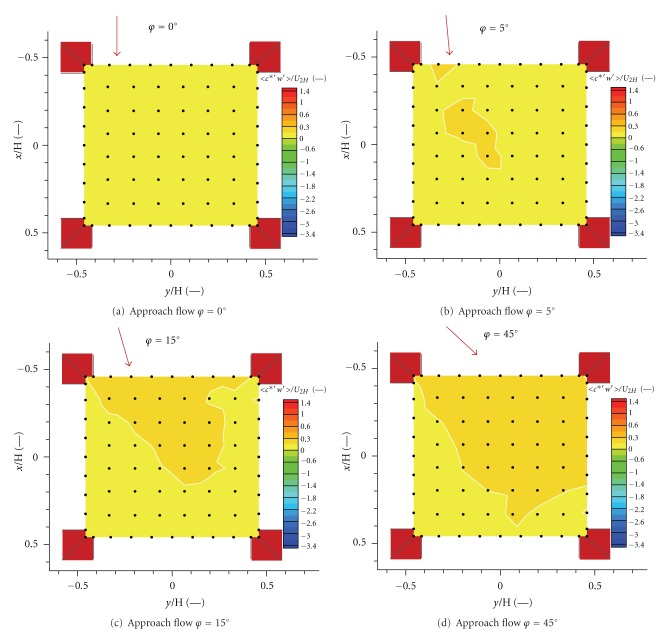
Vertical dimensionless turbulent scalar flux 〈*c* ^∗′^ *w*′〉/*U*_2*H*_ for four angles of the approach flow direction.

Values of determined vertical turbulent fluxes for the four approach flow directions are plotted in Figure 10. We measured relatively flat turbulent flux field by angle *φ* = 0°, but, compared with the advective flux, there is a positive turbulent transport of pollution, compare Figures 10(a) and 9(a).

In case *φ* = 15°, there are significantly positive values on the upwind side of the area (Figure 10(c)). The observed phenomenon became stronger by angle *φ* = 45° (Figure 10(d)).

We estimated a significant turbulent transport of pollution near the leeward side of the buildings, see the upper part of Figures 10(a) and 10(b).

In comparison with the advective transport, the turbulent fluxes are positive in every case. The turbulent fluxes magnitude achieved almost two times the advective fluxes magnitude in the roof top level plane above the studied intersection.

### 3.5. Quadrant Analysis

 We focused on the turbulent flow in vertical direction situated in the horizontal plane at the roof top level above the intersection in this part.

The first step to investigate the turbulent processes in strongly turbulent flow is the quadrant analysis, Kellnerová et al. [26], Feddersen [27]. We applied this analysis to the velocity fluctuation time series to obtain contributions of the vertical flux of longitudinal momentum 〈*u*′*w*′〉 from particular quadrants defined as

1st quadrant “outward interaction" (*u*′ > 0, *w*′ > 0),
2nd quadrant “sweep" (*u*′ > 0, *w*′ < 0),
3rd quadrant “inward interaction" (*u*′ < 0, *w*′ < 0),
4th quadrant “ejection" (*u*′ < 0, *w*′ > 0).

**Figure 11:**
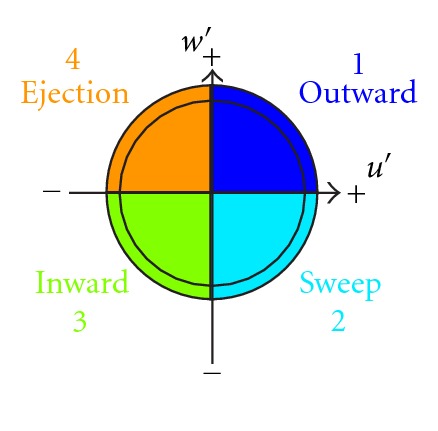
The scheme of event definitions used in velocity fluctuation quadrant analysis.

These definitions are illustrated by Figure 11. The particular contribution from *i*th quadrant to the total momentum flux 〈*u*′*w*′〉 is given by

(7) $$S_{i}=\frac{{\langle u^{\prime }w^{\prime }\rangle }_{i}N_{i}}{N_{\text{total}}},$$

where 〈*u*′*w*′〉_*i*_ is the average stress and *N*_*i*_ is the number of events in the *i*th quadrant, number of all measured events is *N*_total_.

The relative contribution *R* of the prevailing event to the total momentum flux is given by

(8) $$R=\frac{S_{\max}}{\sum S_{i}}100\%,$$

where *S*_max⁡_ is the particular contribution from the dominant event.

**Figure 12:**
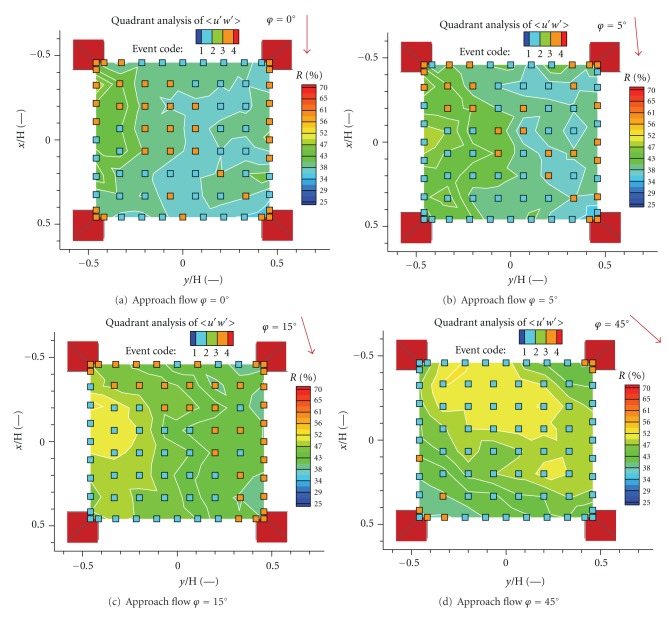
Relative contributions *R* of dominant event to the total momentum flux 〈*u*′*w*′〉 for four angles of the approach flow direction.

Relative contributions *R* of dominant events for four angles of the approach flow directions are plotted in Figure 12. As you see, ejections and sweeps are the prevailing events. Ejections characterize the upward transport of longitudinal momentum deficit, sweeps correspond to the downward transport of longitudinal momentum excess.

Ejections and sweeps were detected for the approach flow direction *φ*≲5° with relatively small relative contribution to the mean momentum flux (Figures 12(a) and 12(b)). Large areas of sweeps with high contribution increased for higher angles *φ* ≳ 15° caused probably by increasing magnitude of longitudinal velocity (Figures 12(c) and 12(d)).

We applied described quadrant analysis to the synchronized vertical velocity and concentration signals. In this case, particular quadrants are defined as

1st quadrant “outward interaction" (*c*′ > 0, *w*′ > 0),
2nd quadrant “sweep" (*c*′ > 0, *w*′ < 0),
3rd quadrant “inward interaction" (*c*′ < 0, *w*′ < 0),
4th quadrant “ejection" (*c*′ < 0, *w*′ > 0).

**Figure 13:**
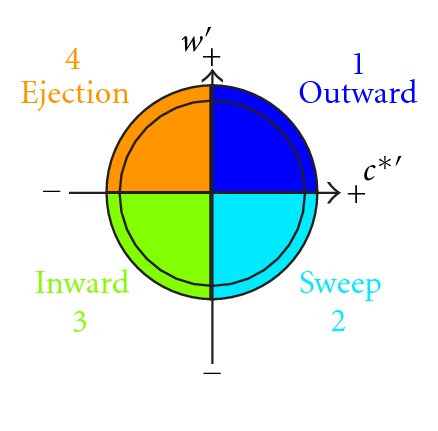
The scheme of event definitions used in turbulent flux quadrant analysis.

These definitions are illustrated by Figure 13.

**Figure 14:**
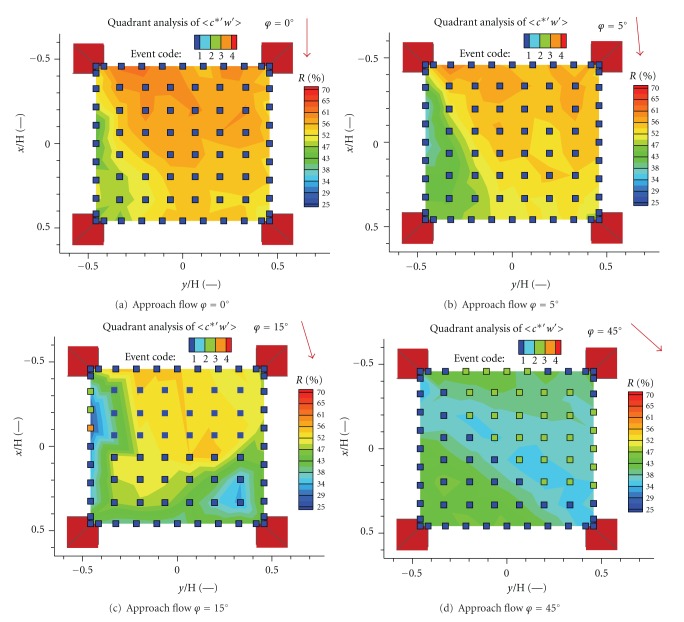
Relative contributions *R* of dominant event to the vertical turbulent scalar flux 〈*c* ^∗′^ *w*′〉/*U*_2*H*_ for four angles of the approach flow direction.

Relative contributions *R* of dominant events for four angles of the approach flow directions are plotted in Figure 14. We observed outward interactions as the dominant event with high relative contribution for the approach flow angles *φ*~0°–15° (Figures 14(a)–14(c)). Inward interaction became dominant in part of the grid for the approach flow angles *φ*~45° but with low relative contribution (Figure 14(d)).

## 4. Conclusion

 The described wind tunnel experiment quantified traffic pollutant dispersion within the X-shaped intersection in an idealized symmetrical urban area depending on the direction of the approach flow. The tracer gas is emitted into the urban area from the point source simulating “pollution hotspot," the place with higher emission of traffic pollution situated near a junction.

Velocity and concentration measurements were done by the building Reynolds number in the interval of Townsend hypothesis validity. We found out very complex flow and dispersion pattern within street canyons and high sensitivity to the approach flow direction. We determined a significant influence of the street canyon arrangements to the horizontal velocity in lower parts of the canyons at vertical levels of *z*≲0.5*H*. The highest concentration of pollution occurred at the bottom levels of streets.

Computed dimensionless advective scalar fluxes of contaminant showed spreading of pollution mostly within the alongwind street for flow almost parallel to the street canyon with pollution source. Spreading of pollution to the crosswind street down the wind was observed for approach flow diverging from orientation of the street canyon with pollution source. We determined the highest advective fluxes at the bottom parts of the street canyons.

A unique experimental setup for simultaneous measurement of the flow velocity and the tracer gas concentration was designed and assembled, based on Fast-Response Flame Ionisation Detector and Laser Doppler Anemometer. Vertical turbulent scalar fluxes of passive contaminant were computed from obtained synchronized signals for a horizontal plane placed above the intersection.

Vertical turbulent fluxes magnitude reached two times higher magnitude of vertical advective fluxes in individual grid points. Determined vertical turbulent fluxes comprised significant and positive contribution to the vertical ventilation of the area. On the other side, horizontal advective fluxes magnitude reached up to four times higher magnitude of vertical turbulent flux, so the contribution of the horizontal advective pollution transport to total ventilation is dominant in all the cases.

The best dispersive conditions in the studied intersection were measured for the approach flow angle *φ* ≈ 15°. In this case we measured generally the lowest concentration in the studied area and the lowest scalar flux from the source to the intersection.

The quadrant analysis was applied to the velocity fluctuation signals determining the sweep as a dominant event in flow above the intersection. The relative contribution of the sweep invents to the momentum flux increased for approach flow diverging from orientation of the street canyon with pollution source.

The quadrant analysis was applied to the synchronized vertical velocity and concentration signals. We determined the outward interaction as a dominant event with high relative contribution to the vertical turbulent flux for flow almost parallel to the street canyon with pollution source. Inward interaction events became dominant for diverging flow but with small relative contribution. The flow in this case is strongly turbulent so that we investigated almost the same contribution to the vertical turbulent flux from all events.

The data set acquired from the experiment in the complex urban structure can be used for validations of numerical models of flow and dispersion in street scale or for comparisons of results obtained using these models. The data contains unique synchronized flow velocity and pollution concentration fluctuations signals in a high temporal resolution that can be used to verify pollution transport properties.

## Figures and Tables

**Table 1:** Parameters of modelled boundary layer above the measured area (in full scale).

*z*_0_ [m]	*d*_0_ [m]	*α* [−]	*u*_∗_/*U*_2*H*_ [−]
0.83	13.40	0.24	0.096
